# The anxious aspects of insecure attachment styles are associated with depression either in pregnancy or in the postpartum period

**DOI:** 10.1186/s12991-020-00301-7

**Published:** 2020-09-09

**Authors:** Emanuela Bianciardi, Cristina Vito, Sophia Betrò, Alberto De Stefano, Alberto Siracusano, Cinzia Niolu

**Affiliations:** 1 Psychiatric Chair, Department of Systems Medicine, University of Rome “Tor Vergata”, Via Cracovia, 50, 00133 Rome, Italy; 2 Volunteers Association of Fondazione Policlinico “Tor Vergata”, Rome, Italy

**Keywords:** Perinatal depression, Attachment style, Life-stress event, Gender medicine, Women health

## Abstract

**Background:**

Perinatal depression (PND) is a major complication of pregnancy and many risk factors have been associated with its development both during pregnancy and postpartum. The transition to motherhood activates the attachment system. The aim of our study was to investigate the relationship between women’s attachment style (AS) and PND in pregnancy, and 1 month after childbirth, in a large cohort of women. We hypothesized that different patterns of AS were associated with either antenatal or postnatal depression. We, further, explored the role of other possible risk factors such as life-stress events.

**Methods:**

A final sample of 572 women was enrolled. At the third trimester of pregnancy, clinical data sheet and self-report questionnaires (ASQ, PSS, LTE-Q, and EPDS) were administered. One month after delivery, EPDS was administered by telephone interview.

**Results:**

We found 10.1% of the women with depression during pregnancy and 11.1% in the postpartum period. The first logistic regression showed that ASQ-CONF subscale (OR = 0.876, *p* < 0.0001), ASQ-NFA subscale (OR = 1.097, *p* = 0.002), foreign nationality (OR = 2.29, *p* = 0.040), low education levels (OR = 0.185, *p* = 0.012), PSS total score (OR = 1.376, *p* = 0.010), and recent life adversities (OR = 3.250, *p* = 0.012) were related to EPDS ≥ 14 during pregnancy.

The second logistic regression showed that ASQ-PRE subscale (OR = 1.077, *p* < 0.001) and foreign nationality (OR = 2.88, *p* = 0.010) were related to EPDS ≥ 12 in the postpartum period.

**Conclusions:**

Different dimensions of anxious insecure AS were, respectively, associated with either antenatal or postnatal depression. These findings support the literature investigating subtypes of perinatal depression. The PND may be heterogeneous in nature, and the comprehension of psychopathological trajectories may improve screening, prevention, and treatment of a disorder which has a long-lasting disabling impact on the mental health of mother and child. We provided a rationale for targeting an attachment-based intervention in this group of women.

## Background

Perinatal depression (PND) is a major complication in pregnancy and the postpartum period, affecting, as many as, one in seven women [1]. This disorder represents a challenge for affected women and clinicians alike, due to its morbidity and potential repercussions for maternal health, fetal development, and child outcomes [2].

Various risk factors have been associated with the development of depression during pregnancy and in the postpartum period including younger age, a history of depression, intimate partner violence, unintended pregnancy, and childhood trauma [3]. The transition to motherhood is a dynamic process developing during pregnancy and after childbirth and it is conceived as a life event that may, in itself, cause considerable distress [4]. Throughout pregnancy and beyond, the transition to motherhood may overwhelm women forced to reestablish their identities in new role [5]. Moreover, motherhood may arouse in women memories of childhood experiences with their own mother’s parenting style and their relationship with caregivers [6]. Clearly, the transition to motherhood triggers the attachment system [7].

The attachment behavioral system is innate and may support individuals in time of need leading her/him to seek the proximity of people who care (i.e. attachment figures). According to the Bowlby theory, the attachment system varies from individual to individual, depending on which particular attachment style (AS) developed, through relationships with caregivers, in the early years of their life [8]. Memories of early experiences with attachment figures are stored as *internal working models* (i.e. mental representations) of self and others. Internal working models incorporate expectations of the self as being/not being worthy of love, and of others as being sure/unsure of providing support in time of need [9]. These working models result in a relatively stable adult attachment style [10]. Thus, adult AS is characterized by a pattern of expectations, needs, emotions, and behaviors in close relationships, which shapes the interpersonal functioning from early life attachment experiences through to adulthood [11]. Moreover, AS is a construct which relates to the attitude of building close relationships that influence emotion regulation and management of life stressors [12].

Securely attached individuals are confident about the support of others and display valid affect-regulation strategies. For example, when the attachment figures are not available, those with secure AS may provoke a mental representation of others with the ability to help them overcome difficult times [13]. In fact, a secure attachment style helps the person to cope with life threats and is known to protect him from psychopathology [14]. By contrast, insecure AS which features either worry about the availability and support of others or distrust and avoidance of intimacy, is a risk factor for the development of various diseases such as eating and somatic disorders and major depression [15, 16]. Surprisingly, although an increasing number of studies showed an association between adult AD and mood disorders [17], the relationship between adult attachment style and depression associated with childbirth has been examined by only a few authors. This emerging body of literature suggested an important link between the insecure attachment style of the mother and the increased incidence of depression in the perinatal period [18, 19].

The aim of our study was to investigate the relationship between women AS and PND in pregnancy and 1 month after birth in a large cohort of women. We hypothesized that different patterns of AS were associated with either antenatal or postnatal depression. We further explored whether AS was associated with PND independent of other possible issues, such as demographic factors, being a first-time mother or recent experience of either stress events or perceived stress events.

## Materials and methods

The data of this study were collected as part of a larger longitudinal study of PND and infant development, which was advanced by the University of Rome “Tor Vergata” and it was promoted by the non-profit Volunteers Association of Tor Vergata Hospital organization. These data come from an arm of the study that was conducted with the cooperation of the Catholic University of Tirana “Our Lady of Good Council” in Albania. From July 2012 to January 2015, a final sample of 572 women was enrolled at the Department of Gynecology and Obstetrics of the Hospital of Durres (Albania) and at the Department of Gynecology and Obstetrics of the two local centers Casilino and Sandro Pertini Hospitals of Rome (Italy) that were affiliated with the University of Rome “Tor Vergata”. The inclusion criteria were being a woman over the age of 18 and in the third trimester of pregnancy. The exclusion criteria were the diagnosis of psychotic spectrum disorders and poor knowledge of language or other limit to verbal communication which compromised the ability of the woman to perform the research protocol.

The study was performed in accordance with the Helsinki declaration standards and was approved by the local Institutional Ethics Review Committee. All the participants provided written informed consent.

There were two phases of the study. In the first step (T0), women were enrolled at the third trimester of pregnancy. A clinical data sheet via face-to-face interview and self-report questionnaires was administered. One month after delivery (T1), EPDS was administered by a telephone interview.

### Socio demographic data sheet

This series of questions was selected on the basis of biography and included age, education, marital status, nationality, employment status, personal and family history of psychiatric disorders. The clinical data sheet was administered by two residents in psychiatry with training relevant to perinatal depression.

### Edinburgh Postnatal Depression Scale

The Edinburgh Postnatal Depression Scale (EPDS) is a 10-item instrument that was developed as a screening tool for postpartum depression and is widely adopted for PND. Each item is scored from 0 to 3 and total scores range from 0 to 30 with higher scores indicating a more severe depression. A score of 14 or higher in pregnancy and of 12 or higher postpartum, has optimal sensitivity and specificity in detecting clinically relevant PND [20–21] We used the Italian validated version of the EPDS [22]. The EPDS t0 and t1 showed internal consistency (Cronbach’s alpha 0.77 and 0.76, respectively).

### Attachment Style Questionnaire

The Attachment Style Questionnaire (ASQ) is a 40-item self-report questionnaire designed to measure dimensions of adult attachment that are central to Hazan and Shaver’s [23] and Bartholomew’s [24]conceptualizations of attachment. The items explore dimensions of attachment theory related to dependence, trust, and self-reliance in close relationships. Participants are instructed to think about their close relationships (whether romantic or not) when answering the questions. Each item is rated on a 6-point scale ranging from 1 (“totally disagree”) to 6 (“totally agree”). The scoring provides five subscales: ASQ Confidence in Self and Others–CONF (eight items), Discomfort with Closeness-DIS (ten items), the Need for Approval-NFA (seven items), Preoccupation with Relationships-PRE (eight items), and Relationships as Secondary-RSA (seven items).

The ASQ has shown adequate reliability [25], with Cronbach’s alpha coefficients for the five scales ranging from 0.81 to 0.87. In the current study, the Italian version of this measure was used [26]. The Cronbach’s alpha values of the ASQ subscales demonstrated good internal consistency (CONFidence: 0.78; DIScomfort with closeness: 0.75; Relationship As Secondary: 0.76; Need For Approval: 0.73; and PREoccupation with relationships: 0.77).

### Last year life events

The List of Threatening Experiences (LTE-Q) [27] is a 12-item brief tool that was used to explore the presence of a life adversity in the previous 12 months: e.g., serious health conditions or death of a family member, significant concerns for a family member, separation or divorce, serious economic concerns, or problems with the law (one or more events vs. none). The LTE-Q was recommended for research purposes in the field of psychiatry and social science [28]and an Italian version was developed [29]. The Cronbach’s alpha was 0.72.

### Perceived Stress Scale

The Perceived Stress Scale is a 14-item self-report questionnaire measuring “the degree to which situations in one’s life are appraised as stressful” [30]. It assesses the degree to which the individual perceived himself as being stressed and overloaded in the past month. In this study, we use the shortened 10-item scale (PSS-10) that showed good psychometric properties and has been recommended for clinical research [31]. We used the Italian version of PSS [32]. The Cronbach’s alpha was 0.71.

### Statistical analyses

A descriptive statistical analysis was conducted, to investigate the frequency of dichotomous variables and the mean scores of dependent variables related to the target scores. Variables were treated either as continuous (ASQ subscales, PSS total score, educational level and age) or binary (nationality, couple relationship, employment, being first-time mothers, previous abortion, preterm birth, history of psychiatric disorder and life adversities in the previous 12 months). The Student's *t* test was used to analyze the mean differences between ASQ subscales and PSS total score in EPDS ≥ 14/EPDS < 14, EPDS ≥ 12/EPDS < 12 and EPDS ≥ 14/EPDS ≥ 12.

The reliability of the questionnaires was verified on the basis of the analysis of its internal homogeneity. The Cronbach’s alpha was calculated to measure the internal homogeneity. It is believed that the internal homogeneity of a questionnaire is satisfactory when the Cronbach’s alpha is between 0.7 and 0.8 [33]. Since all continuous variables except age and PSS total score were non-normally distributed (skewness ratio not within ± 2), nonparametric methods were applied. Zero-order Spearman correlations were calculated between study variables. Bivariate correlations (Pearson *r* two tailed) were used for age and PSS total score variables. With the aim of measuring correlations with the nominal variable (nationality) we used Cramer's *V* test. The Chi-square (*χ*^2^) test was used to determine the significance. An alpha level of 0.05 was used for significance (two tailed). Correlation coefficients are considered to represent a small effect from 0.1 to 0.3, a medium effect from 0.3 to 0.5, and a large effect if greater than 0.5 [34].

We used logistic regression analysis to evaluate the relationship between the predictor variables, including ASQ subscales and the outcome of T0 EPDS ≥ 14 or EPDS < 14/T1 EPDS ≥ 12 or EPDS < 12, to obtain crude odds ratios (ORs). This statistical method was chosen to analyze the effects of independent variables on a binary dependent variable in terms of the probability of being in one of the two categories vs the other. The variables that were correlated with significance level of *p *< 0.05 were included in the logistic regression analysis [35, 36].

Statistical significance level was set, a priori, to *p * < 0.05 and calculations were done with the software IBM SPSS Statistics version 26 for Mac.

## Results

The descriptive statistics of the study sample is illustrated in Table 1. According to the EPDS score, 10.1% of the women (58/572) suffered from depression in pregnancy and 11.1% of them (55/496) in the postpartum period.

**Table 1:** Demographic and psychometric data of study sample (*n* = 572)

Variable	Value	Total no	%	Missed (no.)	% missed
Nationality	Italian/Albanian	442	77.3	0	0.0
	Other	130	22.7		
Mean age (range)		28.8 (15–45) – SD = 5.7			
Education	Primary school	3	0.6	92	16.1
	Junior high school	109	22.7		
	High school	204	42.5		
	Bachelors degree/post-graduate	164	34.2		
Couple relationship	Single/separated/divorced	12	2.2	17	3.0
	Married/cohabiting	543	97.8		
Occupation	Unemployed	220	46.6	100	17.5
	Employed full/part time	252	53.4		
First-time mother	Yes	308	55.4	16	2.8
	No	248	44.6		
Abortion	Yes	199	42.2	100	17.5
	No	273	57.8		
Preterm birth	Yes	70	13.9	69	12.1
	No	433	86.1		
History of psychiatric disorder	Yes	13	2.8	100	17.5
	No	459	97.2		
Mean PSS total score (range)		18.9 (1–38) – SD = 6.6		347	60.7
Recent life adversities (LTE-Q)	Yes	103	28.4	209	36.5
	No	260	71.6		
EPDS ≥ 14 (t0)	Yes	58	10.1		
	No	514	89.9		
EPDS ≥ 12 (t1)	Yes	55	11.1	76	13.3
	No	441	88.9		

*SD* standard deviation

As reported in Table 2, the mean scores of the ASQ subscales (DIS, RAS, NFA, and PRE) and the PSS total score decreased at t0 and t1 (EPDS ≥ 14/EPDS < 14, EPDS ≥ 112/EPDS < 12). The mean score of the ASQ-CONF subscale increased in t0 and t1. At t1, only the mean scores of the ASQ-DIS subscale and the PSS total score were not significant.

**Table 2:** Differences of ASQ and PSS mean scores at t0 and t1

Variable	Value	EPDS ≥ 14		EPDS < 14		*p* value
		Mean	SD	Mean	SD	
ASQ	CONF	30.3	5.3	35.4	5.3	< 0.0001
	DIS	37.6	7.2	34.5	6.9	0.001
	RAS	19.0	5.6	16.2	5.5	< 0.0001
	NFA	22.5	6.9	17.5	5.4	< 0.0001
	PRE	33.0	8.2	28.9	6.4	< 0.0001
PSS total score		23.4	7.3	18.3	6.4	< 0.0001

Variable	Value	EPDS ≥ 12		EPDS < 12		*p* value
		Mean	SD	Mean	SD	
ASQ	CONF	32.9	5.9	35.3	5.4	0.002
	DIS	36.3	6.7	34.7	7.0	0.111
	RAS	18.7	5.9	16.1	5.5	0.001
	NFA	20.5	6.2	17.6	5.7	< 0.0001
	PRE	33.1	6.0	28.7	6.8	< 0.0001
PSS total score		21.2	7.9	18.1	6.6	0.143

*SD* standard deviation

Significative value (*t* test) *p* < 0.05

Bivariate correlations (Pearson r two tailed), Spearman’s correlations, and Cramer’s V test were used to determine whether the women’s ASQ subscales score and predictor variable ratings were significantly related to T0 EPDS ≥ 14. As reported in Table 3, the results demonstrated that the ASQ-CONF subscale was significantly and negatively related to the T0 EPDS ≥ 14 (*r* = − 0.322, *p* < 0.0001). In addition, the ASQ-NFA subscale (*r* = 0.265, *p* < 0.0001) and the PSS total score (*r* = 0.389, *p* < 0.0001) were significantly and positively related to the T0 EPDS ≥ 14.

**Table 3:** Correlations with EPDS in pregnancy

Variable	Value	EPDS ≥ 14 (t0)	
		Correlation	*p* value
ASQ	CONF	− 0.322	< 0.0001
	DIS	0.143	0.001
	RAS	0.182	< 0.0001
	NFA	0.265	< 0.0001
	PRE	0.205	< 0.0001
Nationality^c^		0.085	0.041
Education^a^		− 0.167	< 0.0001
Couple relationship^b^		0.019	0.647
Occupation^b^		0.148	0.001
First-time mother^b^		0.047	0.271
Abortion^b^		0.001	0.975
Preterm birth^b^		0.015	0.742
History of psychiatric disorder^b^		0.061	0.189
PSS total score^a^		0.389	< 0.0001
Recent life adversities (LTE-Q)^b^		− 0.202	< 0.0001

^a^Pearson Correlation (*r*)

^b^Spearman Correlation (rho)

^c^Cramer’s *V* test; Correlation is significant at the 0.01 (two tailed)

However, the ASQ-RAS (*r* = 0.182, *p* < 0.0001), ASQ-PRE (*r* = 0.205, *p* < 0.0001), ASQ-DIS (*r* = 0.143, *p* = 0.001), education (rho = − 0.167, *p* < 0.0001), occupation (rho = 0.148, *p* = 0.001) and recent life adversities (rho = − 0.202, *p* < 0.0001), nationality (*V* = 0.085, *p* = 0.041) for the predictor variables were, only, weakly related to the EPDS cut-off.

Bivariate correlations (Pearson r two tailed) and Spearman’s correlation were used to determine whether the women’s ASQ subscales score and predictor variable ratings were significantly related to T1 EPDS ≥ 12. As reported in Table 4, the results demonstrated that the ASQ-CONF subscale was negatively related to the EPDS ≥ 12 (*r* = − 0.214, *p* < 0.0001). In addition, the ASQ-PRE subscale (rho = 0.260, *p* < 0.0001) and the PSS total score (rho = 0.311 *p* < 0.0001) were significantly and positively related to T1 EPDS ≥ 12.

**Table 4:** Correlations with postpartum EPDS

Variable	Value	EPDS ≥ 12 (t1)	
		Correlation	*p* value
ASQ	CONF	− 0.214	< 0.0001
	DIS	0.162	< 0.0001
	RAS	0.186	< 0.0001
	NFA	0.210	< 0.0001
	PRE	0.260	< 0.0001
Nationality^c^		0.111	0.014
Education^a^		− 0.117	0.017
Couple relationship^b^		0.122	0.007
Occupation^b^		0.081	0.102
First-time mother^b^		0.194	< 0.0001
Abortion^b^		− 0.037	0.457
Preterm birth^b^		0.015	0.743
History of psychiatric disorder^b^		0.076	0.125
PSS total score^a^		0.311	< 0.0001
Recent life adversities (LTE-Q)^b^		− 0.194	0.001

^a^Pearson correlation (*r*)

^b^Spearman correlation (rho)

^c^Cramer’s *V* test; Correlation is significant at the 0.01 (two tailed)

However, the ASQ-RAS (*r* = 0.186, *p* < 0.0001), the ASQ-NFA (*r* = 0.210, *p* < 0.0001), the ASQ-DIS (*r* = 0.162, *p* < 0.0001), education (rho = − 0.117, *p* = 0.017), nationality (*V* = 0.111, *p* = 0.014), couple relationship (rho = 0.122, *p* = 0.007), being first-time mother (rho = − 0.194, *p* < 0.0001) and recent life adversities (rho = − 0.94, *p* = 0.001) were, only, weakly related to the EPDS cut-off.

The correlation methods showed a difference between pregnancy and postpartum period. The effect decreases over time, even if, only, weakly. Only in the ASQ-NFA subscale, nationality and PSS total score, indicated an increase in correlation between T0 and T1. The ASQ-CONF subscale and PSS total score, alone, showed an “average” correlation.

Logistic regressions were conducted to examine the role of all risk factors in modeling EPDS (cut-off score above or equal to 14 and 12, respectively) in two different periods (pregnancy and postpartum). Tables 5 and 6 summarize the results of the logistic regressions.

**Table 5:** Logistic regression (EPDS in pregnancy is the dependent variable)

Variable	Value	EPDS ≥ 14 (t0)		
		*B*	*p* value	Exp (*B*)
ASQ	CONF	− 0.057	< 0.0001	0.876
	NFA	0.040	0.002	1.097
Nationality		0.360	0.0400	2.290
Education		− 0.733	0.012	0.185
PSS total score		0.139	0.010	1.376
Recent life adversities (LTE-Q)		0.512	0.012	3.250

Significative value *p* < 0.05

**Table 6:** Logistic regression (postpartum EPDS is the dependent variable)

Variable	Value	EPDS ≥ 12 (t1)		
		B	*p* value	Exp (*B*)
ASQ	PRE	0.032	< 0.0001	1.077
Nationality		0.459	0.010	2.88
Significative value *p* < 0.05				

The first logistic regression showed that ASQ-CONF subscale (OR = 0.876, *p* < 0.0001), ASQ-NFA subscale (OR = 1.097, *p* = 0.002), foreign nationality (OR = 2.29, *p* = 0.040), low education levels (OR = 0.185, *p* = 0.012), PSS total score (OR = 1.376, *p* = 0.010), and recent life adversities (OR = 3.250, *p* = 0.012) were related to EPDS ≥ 14 during pregnancy.

The second logistic regression showed that ASQ-PRE subscale (OR = 1.077, *p* < 0.001) and foreign nationality (OR = 2.88, *p* = 0.010) were related to EPDS ≥ 12 in the postpartum period.

## Discussion

This study confirmed that PND is a highly prevalent disorder. Pregnancy and the early postpartum phase may be a source of distress. In this delicate phase of life, the insecure attachment style shapes emotions, behaviors and expectations, due to anxiety about close relationships, leading to an increased risk of depression. Moreover, we found that different dimensions of anxious, insecure AS were associated with either antenatal or postnatal depressive symptoms. The *need for approval* pattern, which is characterized by overall low self-confidence and overreliance on others, was found to increase the risk of antenatal depression, while the *preoccupation with relationships* pattern, which is characterized by a lack of confidence either in themselves or in others, was related to postnatal depression. Of the total sample, 10.1% of pregnant women and 11.1% of those who were in the postpartum period showed significant depressive symptoms. The findings are consistent with previous studies reporting the prevalence of PND ranging from 10 to 20% [37]. Among the participants, antenatal depressive symptoms were slightly more prevalent in comparison to the postpartum period. These data are not in accordance with studies documenting a fourfold increased risk of PND in the first month after delivery and a higher prevalence with respect to antenatal depression [38, 39]. It is worth noting that pre-natal women self-reported EPDS, while postnatal women completed the EPDS questionnaire through a telephone interview performed by a clinician. For this reason, we hypothesize that the new mothers may have minimized the severity of depressive symptoms through guilt and fear of stigma [40, 41, 42]. However, telephone screening with EPDS is considered a good alternative to a clinical interview and our results are consistent with data on PND prevalence previously reported [43]. Our main finding was the influence of maternal AS on differential timing of PND. In women with secure AS, depressive symptoms in pregnancy were less likely to develop. In fact, secure AS pregnancy may facilitate mental representations of secure relationships leading to emotion regulation processes. By contrast, the cognitions and behaviors related to insecure AS increased the risk of mood disorder, since the pregnancy was perceived as a source of stress. In particular, as was previously hypothesized, women with inadequate early parenting experiences may get into a psychological crisis when experiencing a healthy pregnancy since they lack a parenting model and are prone to self-criticism and feelings of ineptitude [44].

The ASQ *Need for Approval* subscale, with high anxiety and low avoidance in relationships, corresponds to the anxious attachment style as described by Hazan and Shaver [23]. The individuals with a greater need of approval dimension are characterized by worries about being unworthy of the esteem of others and are inclined to behave in a manner acceptable to other people. They are reported to display beliefs of low self-esteem and poor self-efficacy [45]. A pregnant woman is the sole person taking care of the fetus. Therefore, it is possible that those with a high need of approval dimension of AS may experience the baby in the womb as challenging and surmise that they will be inadequate as mothers. This psychological distress may result in antenatal depression. The ASQ *Preoccupation with Relationships* subscale is consistent with the anxious/ambivalent style conceptualized by Ainsworth [46]. Women with a high score in the ASQ-PRE dimension are characterized by the need to have everything under control, hyper-vigilance to threat-related stimuli, and feelings of anger and anxiety about relationships. In the postpartum phase, the mixture of anxiety, a tendency to depend on others and a lack of self-trust may increase the risk of depression.

Our results are consistent with other lines of research exploring biological, psychological and sociodemographic factors underlying episodes of depression, with onset either during pregnancy or postpartum [47, 48, 49]. From this perspective, we, also, documented that lower education level, being unemployed, perceived stress in the last month and the presence of any recent life adversity raised the probability of suffering from depression during pregnancy as compared to postpartum. We, therefore, confirmed previous data reporting that stressful life events are strongly associated with antenatal depression [50, 51]. With foreign mothers, in particular, we observed an increasing risk of depression when moving from pregnancy to the postpartum period, perhaps due to cultural and affective separation from a familiar support system that might be necessary especially after childbirth [52]. Overall, our findings about AS and PND support the literature searching for subtypes of perinatal depression. It is worth mentioning that PND is heterogeneous in nature and the comprehension of different psychopathological trajectories may improve screening, prevention and treatment of a disorder, with a long-lasting disabling impact on the mental health of mother and child [53]. Ad hoc screening tools and guidelines for different therapeutic needs might be developed classifying the patients according to risk categories and the timing of symptoms. Non-pharmacological interventions may benefit from the AS-based approach and help women to relieve psychological stress resulting from anxiety in close relationships. The AS-based intervention might mitigate maternal depression while indirectly improving mother–infant bonding which is an important mechanism for the transmission of depression to the offspring [54, 55]. Moreover, the advance of our study was to perform a screening for PND during pregnancy and in the postpartum period, assessing crucial risk factors as recent life-stress events and the attachment style [50, 56]. It was suggested that providing the screening among the Departments of Gynecology and Obstetrics may improve the detection of this underrecognized disorder [57]. Starting from these results, we support the need for tailored screening which can give some important insights on the clinical features of women at high risk of depression. Improving the characterization of affected patients may help in establishing flowcharts for treatment pathways in women with risk factors. Although our results along with the treatment implications are compelling, we recognize some limitations. First of all, we could have explored AS using clinical interviews instead of the self-report questionnaire. Even so, the ASQ is a reliable and widely accepted instrument for research purposes [33]. Moreover, we could have compared our data to a sample of parous women with major depression outside the perinatal period. Finally, since the purpose of this study was to perform a screening for perinatal depression [58], women were not submitted neither to a psychiatric evaluation nor to the Structured Clinical Interview for DSM–5 Disorders [59] for making depression diagnosis. Accordingly, we used a validated screening tool as the EPDS that was recommended by international guidelines [60]. Furthermore, we provided contact information about our perinatal health service and all women enrolled in the study agreed to be further approached by our staff in case of any suspected depressive disorder that would come up in the screening.

## Conclusion

To clarify whether a specific AS influenced perinatal depressive symptom is compelling and may improve the screening and the treatment of affected women. We provided a rationale for targeting an attachment-based intervention to this group of women. Studying adult AS in the field of PND may, also, provide subsequent advantages by protecting the offspring from the detrimental effect of maternal depression. Social and psychological risk factors of PND must be further considered.

## Data Availability

The dataset is available from the corresponding author on reasonable request.

## Abbreviations

PND: Perinatal depression
AS: Attachment style
ASQ: Attachment Style Questionnaire
PSS: Perceived Stress Scale
LTE-Q: List of threatening experiences
EPDS: Edinburgh Postnatal Depression Scale
